# Blood Level of 2-arachidonoyl glycerol (2-AG), Neuropeptide Y and Omentin and Their Correlation with Food Habits in Obese Women

**DOI:** 10.31661/gmj.v9i0.1721

**Published:** 2020-12-28

**Authors:** Mozhdeh Rahmanian, Neda Lotfi Yaghin, Mohammad Alizadeh

**Affiliations:** ^1^Student Research Committee, Tabriz University of Medical Sciences, Tabriz, Iran; ^2^Nutrition Research Center, Tabriz University of Medical Sciences, Tabriz, Iran

**Keywords:** Obesity, Omentin, Endocannabinoids, Neuropeptide Y, Feeding Behavior, Fatty Acids

## Abstract

**Background:**

Despite growing concern about the increasing global burden of obesity, there are still many uncertainties in understanding the pathogenesis of this disease. This study aimed to investigate the serum levels of 2-arachidonoylglycerol (2-AG), neuropeptide Y (NPY), and omentin, concerning dietary patterns in obese women.

**Materials and Methods:**

This case-control study was carried on an equal number of obese (case group) and normal-weight women (n=45 each). Dietary intake was determined based on the food frequency questionnaire. Serum levels of 2-AG, NPY, and omentin were determined using ELISA.

**Results:**

The obese group showed significantly higher 2-AG and NPY levels than the controls(P<0.001). There were significant positive correlations between the serum level of 2-AG and calorie intake (r=0.219, P=0.038), carbohydrates (r=0.238, P=0.024), fat (r=0.227, P=0.032), saturated fatty acids (r=0.272, P=0.009), and monounsaturated fatty acids (r=0.265, P=0.012).

**Conclusion:**

Our study revealed that dietary patterns, in particular, the type of fatty acids used may influence levels of 2-AG, NPY, and omentin, which all are involved in pathways resulting in obesity.

## Introduction

Obesity inflicts a well- recognized burden on health [1]. One of the basic principles of nutrition and metabolism is that body weight changes are caused by an imbalance between the energy received from food and the energy used for physical activity and survival [2]. Various central and environmental mechanisms are recognized in regulation of body composition [3]. Different types of hormones, peptides and components/ingredients of tissues and, in particular, the brain contribute to energy balance and metabolism. Among different mechanisms, the endocannabinoid system (ES) has been less investigated. Evidence suggests that cannabinoids are associated with other hormones and adipokines, affecting their secretion and activity. Studies have elaborated cross-linkage between food patterns, especially fat groups and endocannabinoids. Nevertheless, studies on these relationships are limited, and only the effect of specific concentrations of fatty acids (e.g., omega-3) on endocannabinoids have been investigated. ES is recognized as a biological system, which can regulate energy homeostasis through its ligands and receptors in organs, such as adipose tissues. It is composed of fat-derived compounds, including N-arachidonoyl-ethanolamine and 2-arachidonoyl glycerol (2-AG) and receptors, such as cannabinoid receptors 1 (CB1) and CB2 [4]. Anandamide and 2-AG contribute to metabolism and food intake processes through their effects on CBRs [5]. Peptides and metabolic hormones regulate the level of endocannabinoids and modify the expression and activity of CBRs in several tissues [6]. Synthesis of adipokines generated by the adipose tissue is associated with metabolic activities [7]. Neuropeptide Y (NPY), a hypothalamic peptide, an essential food intake and/or energy metabolism regulator [8], is a powerful orexigenic factor hypothalamus, which induces obesity by increasing fat storage and inhibiting the thermogenesis of brown fat tissue. It is also known to induce hyperinsulinemia. Previous findings suggest that the level of NPY is likely to increase in peripheral neurons and adrenergic receptors due to obesity and metabolic diseases by modulating the level of endocannabinoids and CB1 receptors. Moreover, some studies have shown that diseases caused by NPY overactivity can be improved by blocking CB1 receptors [9]. Omentin-1 (34 kDa) is released from adipose tissues, intestinal Paneth and endothelial cells. It plays various roles in the metabolic system, especially by increasing the conduction of insulin signals and transfer of insulin-stimulated glucose in the human adipose tissue; it also participates in the regulation of fat metabolism. Fat cells seem to secret higher levels of omentin than other cells [10]. Since endocannabinoids depend on food-derived fat, a change in diet can change their level [11]. However, the effects of dietary habits on endocannabinoids and NPY are not clarified. Reports have merely described the effect of fat on the level of these components. ES has been found crucial in homeostasis and hedonic appetite, and food intake. In contrast, eating behaviors and patterns directly affect ES activity and other appetite regulators [12]. Thus, it seems necessary to clarify any interactions between ES and neural regulators of appetite with both calorie intake and metabolism. As a first step, we aimed to investigate the serum levels of 2-AG, NPY, and omentin regarding dietary patterns of obese women and elucidate any associations between dietary patterns and the variables mentioned above.

## Materials and Methods

The current case-control research was performed on 90 females, including 45 obese and 45 normal-weight subjects. The sample size was determined according to Yao *et al*. [13], based on the average NPY level at 90% confidence level and 90% power. The case and control groups were recruited from those attending to out-patient departments of Tabriz University of Medical Sciences (TBZMED), Iran, for routine care via convenience accessible sampling. Women with age range of 20-50; body mass index (BMI) of 30 to 39.9 and 18.5-24.9 (case and control groups, respectively) were included. The exclusion criteria were being in the postmenopausal stage, presence of metabolic or malignant diseases, diagnosis of diabetes, thyroid or liver problems, and being on a weight loss diet in the past three months. Ethics panel of Tabriz University of Medical Sciences approved the study (IR.TBZMED.REC.1397.268). The participants were informed about research procedures, and informed consents were obtained prior to the study.

###  Anthropometric Measurements

 Anthropometric assessments were performed by an expert. Height was assessed shoeless through a stadiometer. Weights were determined, and BMI was calculated. Body fat percentage (BF%) was measured by means of a standard body analyzer (Bioassay Technology Laboratory, UK). In addition, waist circumference was evaluated through a measuring tape at the halfway between the lower costal border and the iliac crest. Hip circumference and waist-to-hip ratio (WHR) were also determined. Finally, after 5 min of resting, blood pressure was measured.

###  Blood Sampling and Biochemical Assessments

 After 12-14 hours of fasting, venous blood samples (5 mL) were collected in a completely hygienic laboratory. Each individual’s blood sample was transferred into sterile labeled tubes for separating the serum. The blood samples were spined, and then the serum was frozen immediately until further assessment. The lipid profile and fasting blood sugar (FBS) were assessed using conventional methods. Moreover, the serum levels of 2-AG, NPY, and omentin were evaluated based on enzymatic colorimetric methods through commercially available kits (Shanghai, China) on an automatic analyzer (Stat Fax, Awareness Technology, USA).

###  Questionnaires

 At the beginning of the study, general characteristics of the participants (age, education, and weight loss diet) were evaluated face to face by the researcher. The food frequency questionnaire was applied for determining the dietary habits of the participants and the intake of each food group. All items were entered in the Nutritionist IV software version of 3.5.2 (Databank, San Bruno, CA) to calculate their amount and energy intake.

###  Statistical Analysis

 Analysis performed using SPSS software ver. 22 (IBM, Chicago, IL, USA. The Independent sample t-test was used to evaluate differences between anthropometric and basic characteristics. The serum levels of 2-AG, NPY, and omentin in the two groups were reported as mean ± standard deviation (SD). Moreover, pearson’s correlation coefficient was used for evaluating the associations between study variables and dietary habits at a significance level of 0.05.

## Results

###  Baseline Characteristics and Metabolic Indices

**Figure 1:**
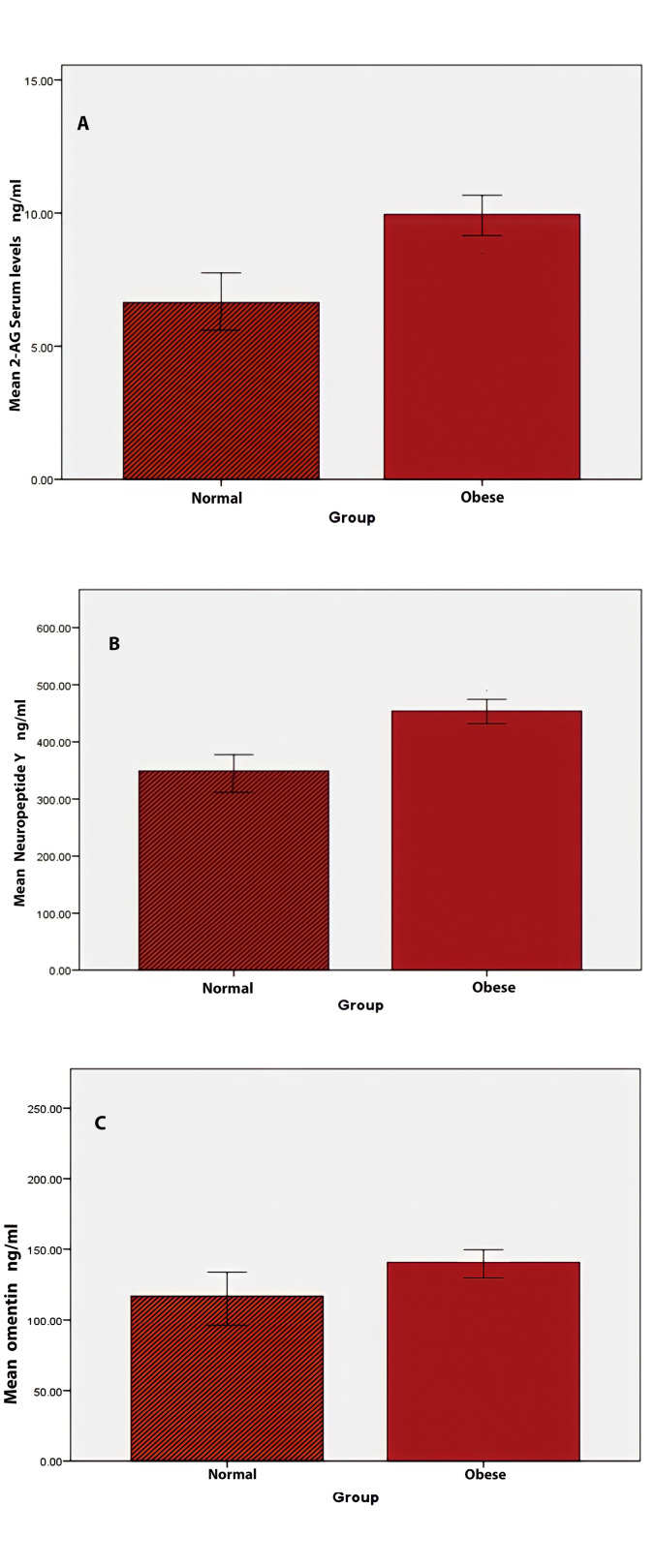
**A:** Mean differences of 2-AG Serum levels (6.64±0.09, 9.94±0.1 and P<0.001), **B:** Mean differences of NP Y (349.31±11.20, 453.97±10.81 and P<0.001) and **C:** Mean differences of omentin (116.93±16.34, 140.68±7.78 and P=0.063) in the case (n=45) and control (n=45) groups

 The subjects’ average age in the control and obese groups was 34.75±7.23 and 33.63±8.09 years, respectively (P=0.498). The mean weight, BMI, WHR, BF%, and BF mass were high in the obese group (P<0.001 for all). FBS was augmented in the obese group and systolic blood pressure (BP, but not diastolic BP), and lower concentrations of high-density lipoprotein (HDL, Table-1). The obese group showed a significantly higher 2-AG level (P<0.001, Figure-1). Also, the case group (453.97±10.8) was found with significantly higher serum NPY level than the controls (P<0.001), while no differences were in the serum level of omentin.

###  Association of Serum 2-AG with NPY and Omentin 

**Figure 2:**
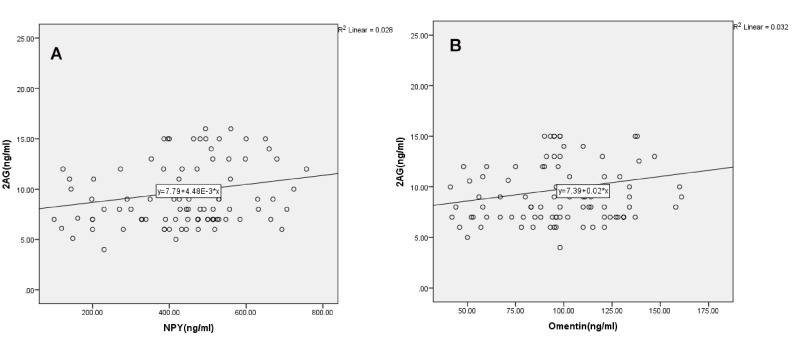
Scatter plot and pearson correlation coefficient (r) of serum 2-AG level with NPY **(A)** and omentin **(B)** in the participants

 According to Figure-2, the serum levels of 2-AG and NPY were significantly correlated (r=0.223, P=0.035). Also, serum levels of 2-AG and omentin showed a positive correlation (r=0.297, P=0.004). In other words, the serum levels of NPY and omentin increased by increasing the level of 2-AG.

###  Correlations of 2-AG, NPY, and Omentin with Dietary Habits 

 We further investigated the correlation of macronutrients (carbohydrates, proteins, fats, and different types of fatty acids) with energy intake. As shown in Table-2, the serum level of 2-AG and energy (r=0.219, P=0.038), carbohydrates (r=0.238, P=0.024), and fat (r=0.227, P=0.032) were found with a positive correlation. Since the relationships of fat groups and fat types were the most significant in this study, we investigated these groups in detail; the results are presented in Table-2. In addition, NPY and fat consumption indicated a moderate correlation (r=0.366, P>0.001).

###  Correlation of 2-AG, NPY, and Omentin with Anthropometric and Biochemical Variables

 The level of 2-AG had a significant positive correlation with weight (r= 0.467, P<0.001), BMI (r=0.536, P<0.001), BF% (r=0.459, P<0.001) and low-density lipoprotein-cholesterol (r=0.235, P=0.027), while it had a negative correlation with HDL (r=-0.308, P=0.004) (Table-3). Moreover, NPY levels were correlated with weight (r=0.350, P=0.001) and BMI (r=0.394, P<0.001), besides some correlations with other factors, listed in Table-3. Our findings revealed no significant correlations between the serum level of omentin and other variables, as shown in Table-3.

## Discussion

The present report determines higher levels of 2-AG and NPY in obese participants, together with augmented NPY production by increasing levels of 2-AG. In this regard, Sitticharoon *et al*. showed a significantly higher mRNA expression of NPY in adipose tissues of obese individuals with higher body fat [14]. Moreover, Cote *et al*. reported higher levels of 2-AG in obese men and found a direct association between 2-AG and BMI [15]. A similar finding was reported by Di Marzo *et al*. [16]. We also assessed the 2-AG, NPY and omentin levels with dietary habits, especially fats, as they can influence the level of endocannabinoids and play a role in the cannabinoid structure. Our findings indicated positive correlations between 2-AG level and energy, carbohydrates, and fatty acids (saturated fatty acids, mono and polyunsaturated FA, oleic and linoleic acids, and linolenic acid). Since our study is the first investigation in this area, we could not find similar articles to investigate 2-AG and other indices, and the results are controversial. In this regard, in an animal study, Artmann *et al*. found that diets rich in arachidonic acid could increase 2-AG in the jejunum [17]. Recently, it has been suggested that a docosahexaenoic acid diet alters the endocannabinoid gene expression and reduces CB1 activation in the muscular cells of treated mice [18]. Kabiri *et al*. suggested that omentin decreased in diet and increased in an olive oil-rich diet [19]. Moreover, Huang *et al*. reported that changes in the amount or quality of dietary fat could significantly alter the hypothalamic expression of neuropeptides, such as NPY [20]. Another study found that long-chain FAS’s hypothalamic concentration could decrease the level of the orexigenic peptides (NPY and agouti-related protein). Consequently, inhibition of food intake and liver glucose production occurs [21]. To date, there are limited human studies investigating the association between type of dietary fatty acids and serum levels of omentin, 2-AG, and NPY. No significant differences in the omentin level between both groups were observed. The serum level of omentin increased by increasing 2-AG, which has not been reported in other studies. De Souza Batista *et al*. showed that omentin and its gene expression decreased due to obesity, and the plasma level of omentin-1 was remarkably attenuated in obese and overweight individuals in comparisons with none-obese participants [22]. Similar observations were documented by O´swiecimska *et al*. and Auguet *et al*. [23,24]. The observed inconsistency between our results and previous studies may be linked to differences in age-grouping gender, physical activity, use of specific drugs, and inflammatory status of the participants. The omentin serum level was also measured, while most previous studies have examined the omentin level of tissues. According to a study by Wilms *et al*., circulating levels of omentin vary with exercise and may increase even if no change occurs in weight [25]. Moreover, Tan *et al*. showed that six months of treatment with metformin could increase the omentin level in women with polycystic ovary syndrome [26]. Although we inquired about the participants’ postmenopausal and hormonal changes, it is suggested to consider exercise and the use of specific drugs in future studies. The present study revealed a clear association between the fatty acid groups with serum level of 2-AG in all participants. However, we did not consider which type of fatty acids could regulate 2-AG, NPY, and omentin levels. If it is confirmed that a specific type of fatty acid can normalize the level of 2-AG, NPY, and omentin, suitable diets could be designed for individuals based on their omentin, NPY, and cannabinoid status. Although it is maybe too early for this proposition, further large-scale human studies can help achieve this goal. In fact, further detailed, large-scale human studies are needed to discover the interactions of these pathways and to propose an appropriate model for effective and sustainable obesity treatment.

## Conclusion

 It is concluded that dietary patterns, in particular type of fatty acids used, may influence the level of endocannabinoids, NPY, and omentin, which all are key regulators of pathways resulting in obesity. Further, more clinical investigations, using a larger sample size, would be of help to more elucidation of associations between each dietary group and neuroendocrine regulation of pathways leading to obesity and possible cross talks with.

## Acknowledgment

 We are grateful to the participants who cooperated with us patiently in this study. We also thank TBZMED for the financial support of this work (grant no:). This study has been conducted as an MSc dissertation of the first author.

## Conflict of Interest

 None.

**Table 1:** Basic Characteristics, Anthropometric Indices and Biochemical Parameters of Study Groups

	**Normal** **N=45**	**Obese** **N=45**	**P**
**Age (years)**	34.75± 7.23	33.63± 8.09	0.498
**Weight (kg)**	59.16± 6.66	80.81± 11.72	<0.001
**BMI (kg/m** ^2^ **)**	22.44± 2.17	31.73± 4.05	<0.001
**WHR (cm)**	0.76± 0.05	0.84± 0.07	<0.001
**BF%**	27.70± 5.05	39.45± 3.7	<0.001
**SBP (mmHg)**	104.52± 9.18	115.50±14.00	<0.001
**DBP (mmHg)**	75.44± 8.40	75.59± 8.48	0.935
**FBS (mg/dl)**	80.11± 6.10	88.36± 6.85	<0.001
**Triglyceride (mg/dl)**	90.84± 35.95	119.43± 50.33	0.003
**Total Cholesterol (mg/dl)**	162.34± 33.16	171.04± 29.13	0.194
**LDL (mg/dl)**	81.37± 26.82	95.97± 24.99	0.010
**HDL (mg/dl)**	62.79± 8.75	51.8± 9.0	<0.001

**BMI: **body mass index; **WHR: **waist/hip ratio; **BF%: **body fat percentage; **SBP:** systolic blood pressure; **DBP: **diastolic blood pressure; **FBS:** fasting blood sugar; ** LDL-C: **low-density lipoprotein-cholesterol; ** HDL-C:** high-density lipoprotein-cholesterol. Values are indicated as mean±SD. The P-values obtained from an independent samples t-test.

**Table 2:** Correlations of the Serum Concentrations of 2-AG, NPY and Omentin with Dietary Habits

	**2-AG**		**NPY**		**Omentin**	
	**r**	**P**	**R**	**P**	**R**	**P**
**Energy (kcal)**	0.219	0.038	0.181	0.088	0.074	0.489
**Protein (g)**	0.147	0.167	0.026	0.808	0.197	0.062
**Carbohydrate (g)**	0.238	0.024	0.095	0.375	0.150	0.158
**TF (g)**	0.227	0.032	0.366	<0.001	0.010	0.922
**SFA (g)**	0.272	0.009	0.354	0.001	-0.069	0.518
**MUFA (g)**	0.265	0.012	0.254	0.016	-0.018	0.863
**PUFA (g)**	0.247	0.019	0.299	0.004	0.000	0.999
**OLA (g)**	0.239	0.023	0.258	0.014	0.020	0.852
**LA (g)**	0.265	0.012	0.361	<0.001	-0.008	0.941
**ALA (g)**	0.241	0.022	0.339	0.001	0.081	0.445
**EPA (g)**	-0.058	0.590	0.119	0.264	0.069	0.521
**DHA (g)**	-0.049	0.647	0.006	0.958	0.077	0.468

**TF:** total fatty acid; **SFA:** saturated fatty acid; **MUFA:** monounsaturated fatty acid; **PUFA:** polyunsaturated fatty acid; **OLA: **oleic acid; ** LA:** linoleic acid; **ALA: **alpha-linolenic acid; **EPA:** eicosapentaenoic acid; **DHA: **docosahexaenoic acid. Data were analyzed by pearson’s correlation test.

**Table 3:** Correlation of 2-AG, NPY, and Omentin Levels with Anthropometric and Biochemical Variables

	**2-AG**		**NPY**		**Omentin**	
	**r**	**P**	**R**	**P**	**R**	**P**
**Weight (kg)**	0.467	<0.001	0.350	0.001	-0.015	0.893
**BMI (kg/m** ^2^ **)**	0.536	<0.001	0.394	<0.001	-0.003	0.980
**WHR (cm)**	0.363	0.001	0.263	0.013	-0.088	0.413
**BF%**	0.459	<0.001	0.339	0.001	-0.010	0.930
**SBP (mmHg)**	0.208	0.052	0.054	0.617	-0.132	0.219
**DBP (mmHg)**	0.100	0.355	0.085	0.431	-0.173	0.107
**FBS (mg/dl)**	0.159	0.138	0.198	0.064	0.054	0.620
**Triglyceride ( mg/dl)**	0.280	0.008	0.208	0.052	-0.227	0.033
**HDL (mg/dl)**	-0.308	0.004	-0.354	0.001	0.093	0.389
**LDL (mg/dl)**	0.235	0.027	0.130	0.227	-0.008	0.941
**Total cholesterol ( mg/dl)**	0.166	0.123	0.057	0.595	-0.032	0.765

**BMI: **body mass index; **WHR: **waist/hip ratio; ** BF%:** body fat percentage; **SBP; **systolic blood pressure; **DBP:** diastolic blood pressure; **FBS;** fasting blood sugar; **HDL-C: **high-density lipoprotein-cholesterol; **LDL-C:** low-density lipoprotein-cholesterol. Data analysis was performed using pearson’s correlation test
